# The Mediating Effects of Diabetes Distress, Anxiety, and Cognitive Fusion on the Association Between Neuroticism and Fear of Hypoglycemia in Patients With Type 2 Diabetes

**DOI:** 10.3389/fpsyg.2021.697051

**Published:** 2021-10-20

**Authors:** Jing Huang, Shenglan Ding, Shuyuan Xiong, Zhiping Liu

**Affiliations:** ^1^Department of Endocrinology, The First Affiliated Hospital of Chongqing Medical University, Chongqing, China; ^2^Department of Nursing, Chengdu Women’s and Children’s Central Hospital, School of Medicine, University of Electronic Science and Technology of China, Chengdu, China

**Keywords:** diabetes distress, anxiety, cognitive fusion, fear of hypoglycemia, mediating effect, type 2 diabetes

## Abstract

**Purpose:** To explore the relationship between neuroticism and fear of hypoglycemia (FoH) among patients with type 2 diabetes (T2D), as well as the mediating effects of diabetes distress, anxiety, and cognitive fusion on the relationship between neuroticism and FoH.

**Methods:** A total of 494 patients with T2D (39.9% females, *n* = 197) were analyzed using the neuroticism scale of the Eysenck Personality Questionnaire-Revised Short Scale (EPQ-RS), the Fear of Hypoglycemia-15 Scale (FH-15), the Diabetes Distress Scale (DDS), the Self-Rating Anxiety Scale (SAS), and the Cognitive Fusion Questionnaire (CFQ). The bootstrapping method was used to test the separate and parallel mediation models.

**Results:** FoH was noted in 17.4% (*n* = 86) of patients. The correlations between neuroticism, diabetes distress, anxiety, cognitive fusion, and FoH were positive. Diabetes distress, anxiety, and cognitive fusion were significant mediators in the association between neuroticism and FoH in both separate and parallel mediation models. In the parallel mediation model, the mediating effect of anxiety was the highest, and the mediating effect of diabetes distress was the lowest, but no significant differences were found in the comparison of these three indirect effects.

**Conclusion:** This study indicated that neuroticism not only directly affected FoH, but also indirectly influenced FoH *via* the increase of diabetes distress, anxiety, and cognitive fusion in patients with T2D. The results provide a theoretical basis for the development of intervention programs to ameliorate patients’ FoH directly and indirectly. Healthcare providers should be encouraged to develop appropriate programs based on improving diabetes distress, anxiety, and cognitive fusion to help patients with T2D improve FoH.

## Introduction

Hypoglycemia occurs frequently in patients with diabetes treated with insulin and/or insulin secretagogues (Fisher et al., 2018; Johnson-Rabbett and Seaquist, 2019). Approximately 25% of patients with type 2 diabetes (T2D) taking insulin for >5 years have severe hypoglycemic episodes, which is the same as the rate of severe hypoglycemic episodes in adults with type 1 diabetes diagnosed within 5 years (UK Hypoglycaemia Study Group, 2007; Heller et al., 2020). The unpleasant symptoms caused by hypoglycemia may make the patients feel frightened; as a result, the patients may maintain high blood glucose levels to prevent hypoglycemia, which may limit the achievement of glycemic control targets (Amiri et al., 2015). The issue of fear of hypoglycemia (FoH) is of great concern worldwide because of its high prevalence and negative influences. In Japan, the prevalence of FoH in insulin-treated patients with T2D was 27.7% (Sakane et al., 2015). FoH not only influences the glycemic control of patients with T2D but also impairs their quality of life (McCoy et al., 2013), resulting in subsequent negative effects on physical and psychological consequences (Benroubi, 2011). Therefore, FoH is one of the crucial factors affecting the psychological health and self-management of patients with T2D. Globally, the number of patients with diabetes was about 463 million in 2019 and will rise to 578 million by 2030, of which T2D accounts for 90% (Zheng et al., 2018; Saeedi et al., 2019). As the prevalence of diabetes continues to rise and the psychological problems of patients with T2D become increasingly serious (Mukherjee and Chaturvedi, 2019; Saeedi et al., 2019), exploring the potential factors associated with FoH is both critical and important.

Neuroticism, one of the five major domains of personality (Goldberg, 1993), refers to the tendency to experience frequent and strong negative emotions when coping with stress, accompanied by a sense of uncontrollability and an insufficient ability to cope (Barlow et al., 2014). Good emotional regulation ability plays a key role in psychological, social and physical health (Yang et al., 2020). However, individuals with stronger neuroticism are more sensitive to negative information and are more susceptible to mental illness (Yang et al., 2020). Moreover, because of the oversensitivity to internal stimuli, people with high neuroticism tend to exhibit exaggerated physical symptoms (Johnson, 2003; Ozer and Benet-Martinez, 2006). The relationship between neuroticism and fear-related disorders has been explored in previous studies and the results showed a significant positive association between neuroticism and fear-related disorders (Ng et al., 2019; Dursun et al., 2021; Perez-Mengual et al., 2021). More specifically, neuroticism has a positive effect on the patient’s tendency to fear about hypoglycemia (Hepburn et al., 1994). The negative tendency of neuroticism may cause patients to pay more attention to hypoglycemia, perceive uncomfortable symptoms more easily, and experience more FoH. However, personality traits tend to be inflexible and stable (Barlow et al., 2014), so more flexible intervention programs are necessary. To the best of our knowledge, few studies have explored the potential mechanisms between neuroticism and FoH.

### The Mediating Role of Diabetes Distress

Diabetes distress refers to the negative emotions or affective experience associated with the challenges of coexistence with diabetes, including different aspects of diabetes management (Fisher et al., 2019; Skinner et al., 2020). Previous studies have found that there is a significant correlation between diabetes distress and FoH (McCarthy et al., 2019; Li et al., 2021). More specifically, the more severe the diabetic distress of the patients, the stronger the FoH. Furthermore, a longitudinal study found that low neuroticism was linked with reduced diabetes distress over the course of a year (Sanatkar et al., 2020), which suggests that diabetes distress may be a specific result of neuroticism. As a result, diabetes distress may be a mediating variable in the relationship between neuroticism and FoH. Based on the findings reported here, we hypothesize (H1) that diabetes distress mediates the relationship between neuroticism and FoH.

### The Mediating Role of Anxiety

Anxiety is a natural emotion of human beings when they have proper warning about an anxiety-producing stimulus, yet anxiety becomes a pathological disorder when it exceeds the normal level and cannot be controlled, requires no special external stimuli, and has a wide range of physical, emotional, behavioral and cognitive changes (Joyce and Herbison, 2015). Several previous studies have shown that there is a strong link between anxiety and FoH (Gonder-Frederick et al., 2006; Anderbro et al., 2015; Rechenberg et al., 2017). An anxiety-prone personality is a predictive factor for FoH (Castellano-Guerrero et al., 2018). Moreover, neuroticism is an anxiety-related endophenotype (Gottschalk and Domschke, 2017) that is positively associated with anxiety (Liao et al., 2019). Based on these findings, we can infer that anxiety may be a mediating variable in the relationship between neuroticism and FoH, which to some extent helps us to propose the hypothesis (H2) that anxiety also mediates the effect of neuroticism on FoH.

### The Mediating Role of Cognitive Fusion

Cognitive fusion, one of the models of the psychopathology of acceptance and commitment therapy (ACT) (Hayes et al., 2006), is a psychological phenomenon in which people believe in the literal meaning of their thoughts instead of regarding thoughts as short internal states (Thomas and Bardeen, 2020). A study reported that cognitive fusion was positively associated with FoH (Wang, 2019). Furthermore, neuroticism is strongly related to psychological inflexibility (Latzman and Masuda, 2013), and cognitive fusion is a component of psychological inflexibility (Hayes et al., 2012). Therefore, cognitive fusion may be a mediating variable in the relation between neuroticism and FoH. Based on this accumulating evidence, in this study, we hypothesize (H3) that cognitive fusion also mediates the effect of neuroticism on FoH.

### Covariates

Factors associated with FoH may influence the relationship between neuroticism and FoH. Female patients have higher FoH scores than male patients (Gjerlow et al., 2014), and older patients are prone to FoH (Anarte et al., 2014; Sakane et al., 2015; Castellano-Guerrero et al., 2018) compared with younger patients. Patients who live alone are also more likely to develop FoH (Sakane et al., 2015). Longer diabetes duration is also linked to greater FoH (Shi et al., 2014). Patients with hypoglycemic episodes in the last year or receiving insulin therapy are at higher risks of FoH (Anarte et al., 2014; Shi et al., 2014). The covariates this study will adjust are age, sex, living alone, duration of diabetes, hypoglycemic episodes in the last year, and duration of insulin use.

Consequently, we adopted separate and parallel mediation models to explore the mediating effects of diabetes distress, anxiety, and cognitive fusion on the relationship between neuroticism and FoH to further clarify the potential mechanisms of neuroticism on FoH, which will provide a theoretical basis for healthcare providers to design intervention programs to improve FoH in patients with T2D.

## Materials and Methods

### Study Design

A cross-sectional study was conducted from July to December 2020. Participants were recruited from the First Affiliated Hospital of Chongqing Medical University, which is a diabetes center in Chongqing, China. Convenience sampling method was used in this study. Structured questionnaires were administered to eligible patients in the inpatient department face to face. All participants signed the informed consent form before the survey, and the research proposal was approved by the Ethics Committee of the First Affiliated Hospital of Chongqing Medical University (2020-418).

### Participants and Procedures

The inclusion criteria were as follows: T2D diagnosed by doctors according to the 1999 World Health Organization criteria, age ≥ 18 years, duration of diabetes ≥ 1 year, and consciousness. The exclusion criteria were as follows: involuntary, pregnancy, condition that was too severe to complete the survey (e.g., headache, palpitations, or dizziness), or a history of mental illness.

For eligible patients, four trained researchers explained the content and aim of the study, and provided the investigation procedure information and the principle of anonymity. Investigations were conducted after the informed consent form was obtained. During the investigation, researchers used uniform instructions to guide and help patients. The researchers collected the questionnaires on the spot upon completion.

### Pre-investigation

We used the first 50 samples as a pre-investigation to calculate the internal consistency of the scales. The internal consistency coefficients of the scale of neuroticism, diabetes distress, anxiety, cognitive fusion, and FoH were 0.853, 0.879, 0.735, 0.915, and 0.951, respectively.

### Data Collection Tools

#### Characteristics of Patients

Sociodemographic and clinical characteristics included age, sex, hypoglycemic episodes in the past year, duration of diabetes, duration of insulin use, and living alone, which were also covariates that were adjusted in the model. Psychological constructs included neuroticism, diabetes distress, anxiety, cognitive fusion, and FoH. Hypoglycemia in patients with T2D was defined as a plasma glucose level ≤3.9 mmol/L. The number of hypoglycemic episodes in the past year came from the patients’ blood glucose monitoring diaries or the patients’ self-reports. Living alone was also self-reported by the patients. Age, sex, duration of diabetes, and duration of insulin use were derived from the electronic medical record system.

#### Eysenck Personality Questionnaire-Revised Short Scale

Neuroticism was evaluated by the validated Chinese version of the neuroticism scale of the Eysenck Personality Questionnaire-Revised Short Scale (EPQ-RS) (Qian et al., 2000). The EPQ-RS was developed by Eysenck et al. (1985) and includes 48 items. Its four domains, namely, neuroticism, extraversion, psychoticism, and a lie scale, respectively, include 12 items. Among them, the items in the neuroticism scale were all scored positively (“yes” = 1, “no” = 0), and the total score range of the neuroticism scale was 0–12. The higher the score, the more prominent the neuroticism personality traits. In our study, the internal consistency coefficient was 0.837.

#### Fear of Hypoglycemia-15 Scale

Fear of hypoglycemia was assessed with the validated Chinese version of the Fear of Hypoglycemia-15 Scale (FH-15) (Liu et al., 2018), which was originally developed by Anarte Ortiz et al. (2011). The FH-15 is a 15-item scale that includes three domains: fear, interference, and avoidance. Each item is rated on 5-point scale, where 1 = “never” to 5 = “every day.” The total score of FH-15 is between 15 and 75. The higher the score, the more serious the fear. In addition, the cutoff score of the Chinese version of the FH-15 is 30.5, which means that patients with a score > 30.5 have FoH. In our study, the internal consistency coefficient was 0.949.

#### Diabetes Distress Scale

The validated Chinese version of the Diabetes Distress Scale (DDS) was used to evaluate diabetes distress (Yang and Liu, 2010). It was developed by Polonsky et al. (2005). The DDS is a 17-item scale with four domains: emotional burden distress, regimen-related distress, diabetes-related interpersonal distress, and physician-related distress. Each item is assessed with a scale of 1 = “no distress” to 6 = “serious distress”. The total score ranges from 17 to 102. The higher the score was, the more severe the distress was. The internal consistency coefficient was 0.915 in the current sample.

#### Self-Rating Anxiety Scale

The Self-Rating Anxiety Scale (SAS) was developed by Zung (1971). In the current study, anxiety was measured with the validated Chinese version of the SAS (Tao and Gao, 1994), which has been widely used in China (Wang et al., 2020). The scale includes 20 items, with each item ranging from 1 = “none, or a little of the time” to 4 = “most, or all of the time.” Among them, the 5th, 9th, 13th, 17th, and 19th items have reverse scoring, i.e., 4 = “none, or a little of the time” to 1 = “most, or all of the time.” The raw score (20–80) was converted to a standard score (25–100) *via* multiplying by 1.25. The higher the score, the greater the anxiety level. The scale showed an internal consistency coefficient of 0.854 in our study.

#### Cognitive Fusion Questionnaire

Cognitive fusion was assessed with the validated Chinese version of the Cognitive Fusion Questionnaire (CFQ) (Zhang et al., 2014). The CFQ was developed by Gillanders et al. (2010), including two subscales of fusion and defusion, with a total of 13 items. The Chinese version of the CFQ only includes the fusion subscale with 9 items, rated on a 7-point scale ranging from 1 = “never true” to 7 = “always true.” The total score of the Chinese version of the CFQ ranges from 9 to 63. The higher the score, the higher the degree of cognitive fusion. In our study, the internal consistency coefficient was 0.936.

### Data Analysis

Statistical analyses were conducted using IBM SPSS Statistics 23.0 and IBM SPSS Amos 23.0. All statistical tests were two-sided, and statistical significance was set as *p*-value < 0.05. Continuous variables with a normal distribution were expressed as the means ± standard deviation (mean ± SD), and the differences between groups were examined by independent-sample t tests. Continuous variables with a skewed distribution were described as the median (interquartile range), and the Mann–Whitney *U* test was used to examine the differences between groups. Categorical variables were presented as frequencies (percentages), and the chi-square test and Fisher’s exact test were used to compare the differences between groups. The Cronbach’s alpha coefficient represented the internal consistency of the scales and composite reliability was also calculated. Spearman coefficients and hierarchical regression were used to assess the correlation between variables.

A bootstrapping method was used to test the mediating effects (MacKinnon et al., 2004; MacKinnon, 2008), because such practice allowed the theory to develop (MacCallum and Austin, 2000; Lin et al., 2020). We performed percentile bootstrapping and bias-corrected percentile bootstrapping at a 95% confidence interval (CI) with 5,000 bootstrap samples, and 95% CI was considered statistically significant if zero was not between the lower and upper bound (Hayes, 2009). Diabetes distress, anxiety, and cognitive fusion were regarded as mediating variables. After adjusting age, sex, duration of diabetes, duration of insulin use, hypoglycemic episodes in the past year, and living alone, we calculated the direct, indirect, and total effects, and we also compared the differences among different indirect effects in the parallel mediation model. The model was considered to have a good fit if χ^2^/df < 3.0, root mean square error of approximation (RMSEA) < 0.06, Tucker-Lewis index (TLI) ≥ 0.95, and confirmatory fit index (CFI) ≥ 0.95 (Hu and Bentler, 1999). In this study, the Mardia’s coefficients for multivariate kurtosis were higher than the cutoff value of 3, indicating that the data deviated from the multivariate normal distribution (Hoi, 2020). Therefore, Bollen–Stine bootstrapping method was performed to evaluate Bollen–Stine *p*-values and Bollen–Stine χ^2^ was used to recalculate the fit indices (Bollen and Stine, 1992).

## Results

A total of 515 questionnaires were distributed, of which 21 questionnaires were uncompleted. Finally, 494 valid questionnaires were included in the analysis.

### Descriptive Analysis

The characteristics of the study participants and a comparison of factors between patients with FoH and patients without FoH are presented in Table 1. The internal consistency coefficients and composite reliability coefficients of the scales are shown in Table 2. The average age of the patients was 60.04 ± 11.71 years, of which 39.9% (*n* = 197) were female. According to the cutoff score of the Chinese version of the FH-15, patients were divided into FoH group and non-FoH group. The prevalence of FoH in patients with T2D in this study was 17.4% (*n* = 86). Compared with male patients, female patients had a higher prevalence of FoH (*p* = 0.035). Patients living alone had a higher rate of FoH than those living with others (*p* = 0.004). Compared with patients in the non-FoH group, patients in the FoH group showed a longer duration of diabetes (*p* = 0.029) and duration of insulin use (*p* < 0.001) and had more hypoglycemic episodes in the past year (*p* < 0.001). Furthermore, the scores of neuroticism, diabetes distress, anxiety, and cognitive fusion in the FoH group were significantly higher than those in the non-FoH group. No significant difference in age was found between the two groups.

**TABLE 1:** Characteristics of the study participants and a comparison of factors between FoH group and non-FoH group.

	Total (*n* = 494)	FoH (*n* = 86)	Non-FoH (*n* = 408)	*p*-value
Sex, *n* (%)				0.035^∗^
Male	297 (60.1)	43 (50.0)	254 (62.3)	
Female	197 (39.9)	43 (50.0)	154 (37.7)	
Age (years), Mean ± *SD*	60.04 ± 11.71	61.30 ± 11.20	59.77 ± 11.81	0.270
Hypoglycemia^a^ (times), Median (*P*_25_, *P*_75_)	1.00 (0.00–3.00)	3.00 (0.75–8.00)	0.00 (0.00–2.00)	< 0.001^∗∗∗^
Duration of diabetes (year), Median (*P*_25_, *P*_75_)	10.00 (5.00–15.00)	12.00 (7.50–17.00)	10.00 (4.00–15.00)	0.029^∗^
Duration of insulin use (years), median (*P*_25_, *P*_75_)	0.00 (0.00, 7.00)	5.00 (0.00–12.00)	0.00 (0.00, 5.00)	< 0.001^∗∗∗^
Living alone, *n* (%)				0.004^∗∗^
Yes	42 (8.5)	14 (16.3)	28 (6.9)	
No	452 (91.5)	72 (83.7)	380 (93.1)	
Neuroticism, median (*P*_2__5_, *P*_75_)	2.00 (0.00–3.25)	3.00 (2.00–6.00)	1.00 (0.00–3.00)	< 0.001^∗∗∗^
Diabetes distress, median (*P*_25_, *P*_75_)	25.00 (21.00–34.00)	34.50 (25.00–43.00)	24.00 (20.00–31.00)	< 0.001^∗∗∗^
Anxiety, median (*P*_25_, *P*_75_)	37.50 (32.50–45.00)	44.38 (38.75–55.00)	36.25 (31.25–42.50)	< 0.001^∗∗∗^
Cognitive fusion, median (*P*_25_, *P*_75_)	23.00 (17.00–33.00)	33.00 (25.75–41.00)	21.00 (16.00–31.00)	< 0.001^∗∗∗^

^*a*^Hypoglycemic episodes in the past year. FoH, fear of hypoglycemia; SD, standard deviation; P, percentile. Significant codes: ***p < 0.001, **p < 0.01, *p < 0.05.

**TABLE 2:** The internal consistency coefficients and composite reliability coefficients of the scales.

	Domain	Loading	Cronbach’s alpha	CR
FH-15			0.949	
	Fear	0.850	0.895	0.896
	Interference	0.876	0.908	0.908
	Avoidance	0.921	0.846	0.849
DDS			0.915	
	Emotional	0.859	0.826	0.830
	Physician	0.662	0.818	0.827
	Regimen	0.800	0.807	0.806
	Interpersonal	0.713	0.814	0.822
EPQ-RS-N	.	.	0.837	0.845
SAS	.	.	0.854	0.868
CFQ	.	.	0.936	0.936

*FH-15, the Fear of Hypoglycemia-15 Scale; DDS, the Diabetes Distress Scale; EPQ-RS-N, the neuroticism scale of the Eysenck Personality Questionnaire-Revised Short Scale; SAS, the Self-Rating Anxiety Scale; CFQ, the Cognitive Fusion Questionnaire; CR, composite reliability.*

### Bivariate Correlation Analysis

The correlation analysis results of neuroticism, diabetes distress, anxiety, cognitive fusion, and covariates are shown in Table 3. Neuroticism was positively correlated with FoH (*r* = 0.364, *p* < 0.001), diabetes distress (*r* = 0.432, *p* < 0.001), anxiety (*r* = 0.553, *p* < 0.001), and cognitive fusion (*r* = 0.567, *p* < 0.001). Diabetes distress (*r* = 0.397, *p* < 0.001), anxiety (*r* = 0.435, *p* < 0.001), and cognitive fusion (*r* = 0.419, *p* < 0.001) were all positively associated with FoH.

**TABLE 3:** Spearman’s correlation matrix of the variables of interest.

	1	2	3	4	5	6	7	8	9	10	11
(1) Sex	1										
(2) Age	0.191^∗∗∗^	1									
(3) Hypoglycemia^a^	0.111^∗^	0.061	1								
(4) Duration of diabetes	0.187^∗∗∗^	0.485^∗∗∗^	0.233^∗∗∗^	1							
(5) Duration of insulin use	0.155^∗∗^	0.212^∗∗∗^	0.402^∗∗∗^	0.526^∗∗∗^	1						
(6) Living alone	–0.011	0.021	0.066	0.057	0.058	1					
(7) Neuroticism	0.130^∗∗^	0.002	0.161^∗∗∗^	0.134^∗∗^	0.057	0.194^∗∗∗^	1				
(8) Anxiety	0.137^∗∗^	0.029	0.179^∗∗∗^	0.145^∗∗^	0.117^∗∗^	0.150^∗∗^	0.553^∗∗∗^	1			
(9) Diabetes distress	–0.008	–0.085	0.102^∗^	0.054	0.042	0.161^∗∗∗^	0.432^∗∗∗^	0.570^∗∗∗^	1		
(10) Cognitive fusion	0.224^∗∗∗^	0.091^∗^	0.161^∗∗∗^	0.098^∗^	0.058	0.148^∗∗^	0.567^∗∗∗^	0.410^∗∗∗^	0.405^∗∗∗^	1	
(11) FoH	0.102^∗^	0.092^∗^	0.378^∗∗∗^	0.164^∗∗∗^	0.202^∗∗∗^	0.086	0.364^∗∗∗^	0.435^∗∗∗^	0.397^∗∗∗^	0.419^∗∗∗^	1

^*a*^Hypoglycemic episodes in the past year. FoH, fear of hypoglycemia. Significant codes: ***p < 0.001, **p < 0.01, *p < 0.05.

### Hierarchical Regression Model

The hierarchical regression models with FoH as the dependent variable and neuroticism, diabetes distress, anxiety, and cognitive fusion as the independent variables are presented in Table 4. In model 2, after adjusting covariates, neuroticism was positively related to FoH (β = 0.328, *p* < 0.001). In model 5, after adjustment, diabetes distress (β = 0.179, *p* < 0.001), anxiety (β = 0.207, *p* < 0.001), and cognitive fusion (β = 0.204, *p* < 0.001) were positively associated with FoH, while the relationship between neuroticism and FoH (β = 0.047, *p* = 0.348) was not significant.

**TABLE 4:** Results of the hierarchy regression analysis.

	Model 1			Model 2			Model 3		
	U. β	S. β	*p*-value	U. β	S. β	*p*-value	U. β	S. β	*p*-value
Sex	1.257	0.072	0.101	0.638	0.037	0.380	1.149	0.066	0.100
Age	0.032	0.044	0.368	0.047	0.064	0.168	0.060	0.083	0.063
Living alone	3.051	0.100	0.021^∗^	1.036	0.034	0.414	0.677	0.022	0.576
Hypoglycemia^a^	0.285	0.224	< 0.001^∗∗∗^	0.257	0.202	< 0.001^∗∗∗^	0.244	0.192	< 0.001^∗∗∗^
Duration of diabetes	–0.049	–0.043	0.472	–0.090	–0.079	0.162	–0.101	–0.088	0.103
Duration of insulin use	0.202	0.135	0.014^∗^	0.214	0.143	0.006^∗∗^	0.216	0.145	0.004^∗∗^
Neuroticism				0.996	0.328	< 0.001^∗∗∗^	0.681	0.224	< 0.001^∗∗∗^
Diabetes distress							0.231	0.294	< 0.001^∗∗∗^
Anxiety									
Cognitive fusion									
*R* ^2^	10.4%			20.4%			27.8%		
Change in *R*^2^	10.4%			10.0%			7.4%		

	**Model 4**			**Model 5**					
	**U. β**	**S. β**	***p*-value**	**U. β**	**S. β**	***p*-value**			

Sex	0.739	0.042	0.285	0.182	0.010	0.792			
Age	0.054	0.074	0.090	0.042	0.058	0.181			
Living alone	0.513	0.017	0.666	0.173	0.006	0.882			
Hypoglycemia^a^	0.234	0.184	< 0.001^∗∗∗^	0.226	0.177	< 0.001^∗∗∗^			
Duration of diabetes	–0.100	–0.087	0.098	–0.084	–0.073	0.161			
Duration of insulin use	0.196	0.131	0.007^∗∗^	0.187	0.126	0.009^∗∗^			
Neuroticism	0.412	0.135	0.004^∗∗^	0.143	0.047	0.348			
Diabetes distress	0.163	0.208	< 0.001^∗∗∗^	0.141	0.179	< 0.001^∗∗∗^			
Anxiety	0.205	0.223	< 0.001^∗∗∗^	0.191	0.207	< 0.001^∗∗∗^			
Cognitive fusion				0.151	0.204	< 0.001^∗∗∗^			
*R* ^2^	30.5%			33.1%					
Change in *R*^2^	2.7%			2.6%					

^*a*^Hypoglycemic episodes in the past year. Age, sex, duration of diabetes, duration of insulin use, hypoglycemic episodes in the past year, and living alone were adjusted. U., unstandardized; S., standardized. Significant codes: ***p < 0.001, **p < 0.01, *p < 0.05.

### Separate Mediation Analysis With Mediator of Diabetes Distress, Anxiety, and Cognitive Fusion

#### Mediating Effect of Diabetes Distress

**FIGURE 1:**
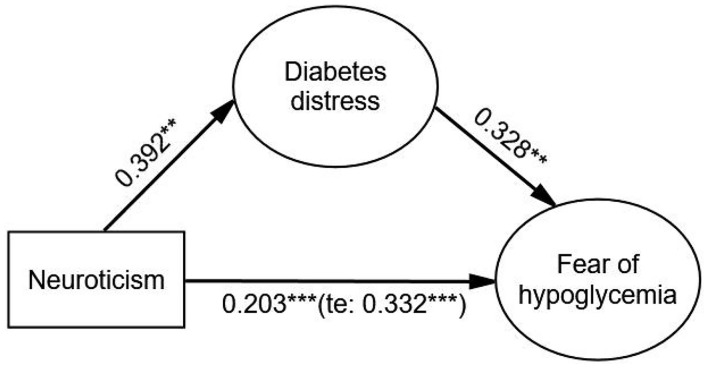
The mediating effect of diabetes distress on the relationship between neuroticism and FoH. Standardized estimating of 5,000 bootstrap sample. Age, sex, duration of diabetes, duration of insulin use, hypoglycemic episodes in the past year, and living alone were controlled. te, total effect. Significant codes: ***p < 0.001, **p < 0.01.

After adjusting covariates, the results showed that neuroticism had a significant effect on FoH (β = 0.332, *p* < 0.001), and the indirect effect of diabetes distress (β = 0.129, *p* < 0.001) on the relationship between neuroticism and FoH was significant. The direct effect of neuroticism on FoH was still significant (β = 0.203, *p* < 0.001) when diabetes distress was added as a mediating variable. The Bollen–Stine adjusted fit indices indicated a good fit: Bollen–Stine bootstrap *p*-value < 0.01, χ^2^/df = 1.331, RMSEA = 0.026, CFI = 0.991, TLI = 0.989. The results suggested that diabetes distress was a partial mediator in the association between neuroticism and FoH (see Figure 1 and Table 5).

**TABLE 5:** Direct, indirect, and total effects of the separate mediation model with mediator of diabetes distress, anxiety, and cognitive fusion.

Mediator	Effect	Path	Point estimate	Bootstrapping				
				Percentile 95% CI		Bias-corrected percentile 95% CI		*p*-value
				Lower	Upper	Lower	Upper	
Diabetes distress	Direct effect	Neuroticism → FoH	0.203	0.082	0.306	0.090	0.311	< 0.001^∗∗∗^
		Neuroticism → diabetes distress	0.392	0.284	0.501	0.278	0.497	0.001^∗∗^
		Diabetes distress → FoH	0.328	0.211	0.454	0.206	0.447	0.001^∗∗^
	Indirect effect	Neuroticism → diabetes distress → FoH	0.129	0.072	0.205	0.070	0.203	< 0.001^∗∗∗^
	Total effect	Neuroticism → FoH	0.332	0.232	0.422	0.237	0.425	< 0.001^∗∗∗^
Anxiety	Direct effect	Neuroticism → FoH	0.150	0.032	0.262	0.037	0.267	0.014^∗^
		Neuroticism → anxiety	0.562	0.493	0.626	0.493	0.627	< 0.001^∗∗∗^
		Anxiety → FoH	0.339	0.226	0.448	0.227	0.449	< 0.001^∗∗∗^
	Indirect effect	Neuroticism → anxiety → FoH	0.190	0.123	0.261	0.125	0.264	< 0.001^∗∗∗^
	Total effect	Neuroticism → FoH	0.341	0.244	0.429	0.248	0.434	< 0.001^∗∗∗^
Cognitive fusion	Direct effect	Neuroticism → FoH	0.184	0.059	0.301	0.064	0.304	0.002^∗∗^
		Neuroticism → cognitive fusion	0.552	0.483	0.616	0.480	0.614	0.001^∗∗^
		Cognitive fusion → FoH	0.283	0.173	0.389	0.171	0.389	< 0.001^∗∗∗^
	Indirect effect	Neuroticism → cognitive fusion → FoH	0.156	0.095	0.220	0.096	0.220	< 0.001^∗∗∗^
	Total effect	Neuroticism → FoH	0.340	0.242	0.429	0.245	0.433	< 0.001^∗∗∗^

Standardized estimating of 5,000 bootstrap sample. Age, sex, duration of diabetes, duration of insulin use, hypoglycemic episodes in the past year, and living alone were adjusted in these three models. FoH, fear of hypoglycemia; CI, confidence interval. Significant codes: ***p < 0.001, **p < 0.01, *p < 0.05.

#### Mediating Effect of Anxiety

**FIGURE 2:**
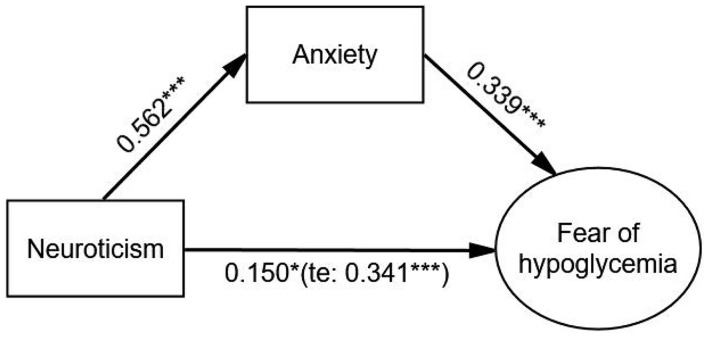
The mediating effect of anxiety on the relationship between neuroticism and FoH. Standardized estimating of 5,000 bootstrap sample. Age, sex, duration of diabetes, duration of insulin use, hypoglycemic episodes in the past year, and living alone were controlled. te, total effect. Significant codes: ***p < 0.001, *p < 0.05.

After adjustment, the indirect effect of anxiety (β = 0.190, *p* < 0.001) on the relationship between neuroticism and FoH was significant, and the direct effect of neuroticism on FoH was weaker but still significant (β = 0.150, *p* = 0.014) compared with the total effect (β = 0.341, *p* < 0.001). The Bollen–Stine adjusted fit indices indicated a good fit: Bollen–Stine bootstrap *p*-value < 0.01, χ^2^/df = 1.210, RMSEA = 0.021, CFI = 0.995, TLI = 0.994. The results revealed that anxiety was a partial mediator in the relationship between neuroticism and FoH (see Figure 2 and Table 5).

#### Mediating Effect of Cognitive Fusion

**FIGURE 3:**
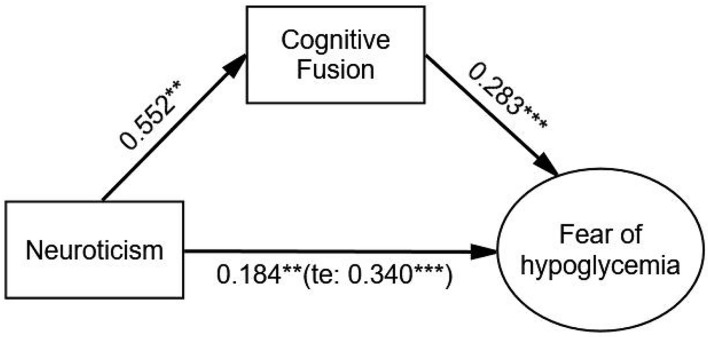
The mediating effect of cognitive fusion on the relationship between neuroticism and FoH. Standardized estimating of 5,000 bootstrap sample. Age, sex, duration of diabetes, duration of insulin use, hypoglycemic episodes in the past year, and living alone were controlled. te, total effect. Significant codes: ***p < 0.001, **p < 0.01.

After adjusting covariates, the relationship between neuroticism and FoH was significantly mediated by cognitive fusion (β = 0.156, *p* < 0.001). Neuroticism had a significant effect on FoH (β = 0.340, *p* < 0.001), and the direct effect of neuroticism on FoH was still significant (β = 0.184, *p* = 0.002) when cognitive fusion was used as a mediating variable. The Bollen–Stine adjusted fit indices indicated a good fit: Bollen–Stine bootstrap *p*-value < 0.01, χ^2^/df = 1.189, RMSEA = 0.020, CFI = 0.996, TLI = 0.994. The results showed that cognitive fusion was also a partial mediator in the relationship between neuroticism and FoH (see Figure 3 and Table 5).

### Parallel Mediation Analysis With Mediator of Diabetes Distress, Anxiety, and Cognitive Fusion

**FIGURE 4:**
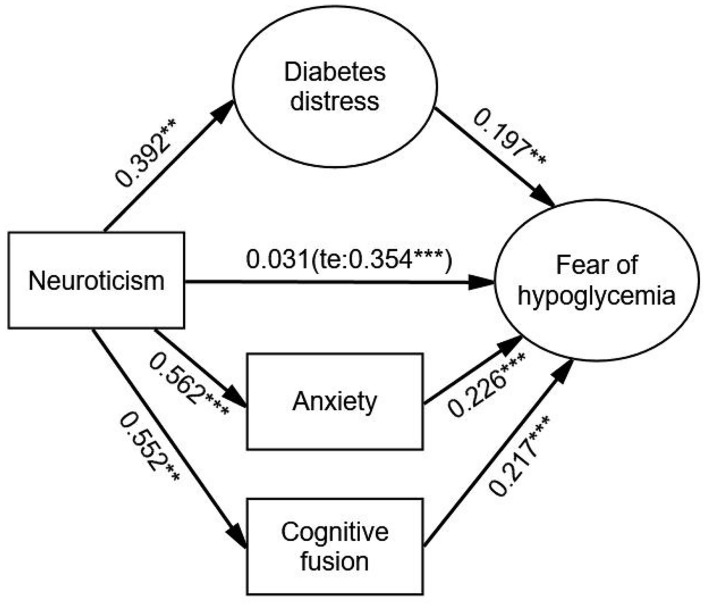
The parallel mediation model with mediator of diabetes distress, anxiety, and cognitive fusion. Standardized estimating of 5,000 bootstrap sample. Age, sex, duration of diabetes, duration of insulin use, hypoglycemic episodes in the past year, and living alone were controlled. te, total effect. Significant codes: ***p < 0.001, **p < 0.01.

After adjustment, diabetes distress (β = 0.077, *p* < 0.001), anxiety (β = 0.127, *p* < 0.001), and cognitive fusion (β = 0.120, *p* < 0.001) had significant mediating effects on the relationship between neuroticism and FoH. The direct effect of neuroticism on FoH was weaker and not significant (β = 0.031, *p* = 0.590) compared with the total effect (β = 0.354, *p* < 0.001). The order of the values of the three indirect effects from large to small were anxiety, cognitive fusion and diabetes distress. There was no significant difference in the comparison of these three indirect effects. The Bollen–Stine adjusted fit indices indicated a good fit: Bollen–Stine bootstrap *p*-value < 0.01, χ^2^/df = 1.308, RMSEA = 0.025, CFI = 0.991, TLI = 0.989. The results suggested that diabetes distress, anxiety, and cognitive fusion jointly and fully mediated the association between neuroticism and FoH (see Figure 4 and Table 6).

**TABLE 6:** Direct, indirect, and total effects of the parallel mediation model with mediator of diabetes distress, anxiety, and cognitive fusion.

Path	Point estimate		Bootstrapping			
		Percentile 95% CI		Bias-corrected percentile 95% CI		*p*-value
		Lower	Upper	Lower	Upper	
**Direct effect**						
Neuroticism → FoH	0.031	–0.103	0.153	–0.093	0.163	0.590
Neuroticism → diabetes distress	0.392	0.283	0.501	0.277	0.496	0.001^∗∗^
Neuroticism → anxiety	0.562	0.493	0.626	0.493	0.627	< 0.001^∗∗∗^
Neuroticism → cognitive fusion	0.552	0.483	0.616	0.480	0.614	0.001^∗∗^
Diabetes distress → FoH	0.197	0.084	0.332	0.080	0.326	0.001^∗∗^
Anxiety → FoH	0.226	0.103	0.347	0.106	0.352	< 0.001^∗∗∗^
Cognitive fusion → FoH	0.217	0.101	0.323	0.101	0.323	< 0.001^∗∗∗^
**Indirect effect**						
Neuroticism → diabetes distress → FoH	0.077	0.030	0.146	0.029	0.144	< 0.001^∗∗∗^
Neuroticism → anxiety → FoH	0.127	0.055	0.203	0.059	0.205	< 0.001^∗∗∗^
Neuroticism → cognitive fusion → FoH	0.120	0.055	0.183	0.057	0.184	< 0.001^∗∗∗^
**Total indirect effect**						
Neuroticism → FoH	0.324	0.238	0.418	0.235	0.416	< 0.001^∗∗∗^
**Total effect**						
Neuroticism → FoH	0.354	0.254	0.443	0.260	0.449	< 0.001^∗∗∗^
**Difference of specific indirect effects**						
Diabetes distress-anxiety	–0.050	–0.153	0.065	–0.156	0.060	0.354
Anxiety-cognitive fusion	0.008	–0.088	0.102	–0.087	0.103	0.864
Diabetes distress-cognitive fusion	–0.042	–0.129	0.063	–0.130	0.061	0.380

Standardized estimating of 5,000 bootstrap sample. Diabetes distress, anxiety, and cognitive fusion were parallel mediating variables. Age, sex, duration of diabetes, duration of insulin use, hypoglycemic episodes in the past year, and living alone were adjusted. FoH, fear of hypoglycemia; CI, confidence interval. Significant codes: ***p < 0.001, **p < 0.01.

## Discussion

To our knowledge, this is the first study to perform structural equation model to reveal whether the relationship between neuroticism and FoH is mediated by diabetes distress, anxiety, and cognitive fusion. The separate and parallel mediation analyses conducted by the bootstrapping method supported our research hypotheses. The results of this study substantiated the significant mediating role of diabetes distress, anxiety, and cognitive fusion in the relationship between neuroticism and FoH. Moreover, in the parallel mediation model, these three constructs were full mediators; among them, anxiety had the highest level of indirect effects, followed by cognitive fusion and diabetes distress.

These three mediators were conducive to explaining the mechanism of why neuroticism may be one of the contributors to FoH. First, the results of the current study revealed that diabetes distress mediated the relationship between neuroticism and FoH. In this model, neuroticism was related to a high risk of diabetes distress and diabetes distress was associated with high risks of FoH, which was in line with previous studies (Sanatkar et al., 2020; Li et al., 2021). Patients with diabetes need to adhere to hypoglycemic treatments and a series of management methods, such as diet control, for a long time due to the chronic and long-term nature of diabetes, which can easily give rise to negative emotions in patients (Skinner et al., 2020). Moreover, functional magnetic resonance imaging (fMRI) results showed that neuroticism scores were inversely related to the activation of some brain regions during negative emotion regulation, indicating that neuroticism weakens one’s ability to regulate emotions (Yang et al., 2020). More specifically, patients with high neuroticism are even more unable to regulate negative emotions when coping with diabetes and tend to produce a sense of powerlessness and distress, which may ultimately lead to fear-related disorders such as FoH (Fisher et al., 2019). Therefore, a potential explanation for the positive correlation between neuroticism and FoH is that patients with high levels of neuroticism may develop FoH due to the high risks of diabetes distress. Interventions aimed at improving neuroticism and diabetes distress should be provided for patients with T2D due to the mediating effects of diabetes distress on the relationship between neuroticism and FoH.

Second, we found that anxiety was a significant mediator in the relationship between neuroticism and FoH. A previous study found that there was an overlap between the genetic factors that affect the variation of neuroticism among individuals and those that increase anxiety (Hettema et al., 2004). The vulnerability model claims that personality is always formed before the development of mental illness (Kotov et al., 2010). In other words, neuroticism indicates an anxiety-prone personality (Brandes and Bienvenu, 2006). This neuroticism-anxiety orientation is consistent with our results that neuroticism has a positive predictive effect on anxiety. Furthermore, increased anxiety can trigger defensive and maladaptive behaviors in patients, leading to clinical problems (Grecucci et al., 2020). For example, patients with FoH tend to show avoidance behaviors, including cutting the dose of insulin, overeating, and reducing physical activity, etc. As shown in several studies, the relationship between anxiety and FoH was significant (Gonder-Frederick et al., 2006; Anderbro et al., 2015; Rechenberg et al., 2017; Castellano-Guerrero et al., 2018), and a study regarded FoH as one of the anxiety-related syndromes associated with diabetes (Majidi et al., 2015). Consequently, patients with anxiety may be more susceptible to FoH. Another potential explanation for the positive correlation between neuroticism and FoH is that patients with high neuroticism personality traits may be more prone to anxiety, and the negative effects caused by anxiety may further increase one’s risk of FoH. Our results indicate that anxiety is also a useful intervention target for FoH in patients with T2D.

Third, the present study showed that neuroticism indirectly increased FoH through cognitive fusion. Some studies showed a significant correlation between neuroticism and psychological inflexibility (Latzman and Masuda, 2013; Paulus et al., 2016), which was in line with our results that neuroticism was significantly positively related to cognitive fusion. People with neuroticism traits are more likely to perceive negative information, and cognitive fusion makes people susceptible to these negative emotions. Further, we also found that cognitive fusion was significantly related to FoH, which was aligned with previous research (Wang, 2019). This may mean that cognitive fusion prompts patients to automatically extract the literal meaning of negative thinking and produce incorrect self-cognition, thereby promoting or aggravating the fear and worry of hypoglycemia. Consequently, the third potential explanation for the positive correlation between neuroticism and FoH is that patients with high neuroticism may be more susceptible to cognitive fusion, thereby increasing their risks of FoH. These results further indicate that interventions aimed at improving cognitive fusion may contribute to the improvement of FoH.

The main strength of this study is to explore the mediating role of three potential psychological factors containing diabetes distress, anxiety, and cognitive fusion in the relationship between neuroticism and FoH. In terms of clinical implications, this study offers an understanding of the associated factors that may contribute to ameliorate patients’ FoH and provides a theoretical basis for healthcare providers to take measures to directly and indirectly improve FoH *via* improving neuroticism, diabetes distress, anxiety, and cognitive fusion. The evaluation tools used in this study have sufficient internal consistency coefficients and composite reliability coefficients in this sample, indicating that the results are highly reliable. Despite such strengths, our study also has several limitations. First, the cross-sectional design limits the ability to explain strong causality. Therefore, future studies should adopt longitudinal designs. Second, the study only recruited patients in China. There may be cultural differences among patients in different countries; therefore, future studies need to investigate whether patients in different countries also fit the same mediation model. Third, the questionnaires were all self-reported in nature, which may lead to social desirability bias and memory recall bias. Finally, this study only investigated patients with T2D; thus, future studies should verify whether patients with type 1 diabetes experience the same mediating effects.

## Conclusion

This study demonstrates that diabetes distress, anxiety, and cognitive fusion are important mediators in the relationship between neuroticism and FoH in patients with T2D. The results provide a theoretical basis for the development of intervention programs to ameliorate patients’ FoH directly and indirectly. Healthcare providers may gain implications from these findings and focus more on improving neuroticism, diabetes distress, anxiety, and cognitive fusion to ameliorate FoH.

## Data Availability Statement

The raw data supporting the conclusions of this article will be made available by the authors, without undue reservation.

## Ethics Statement

The studies involving human participants were reviewed and approved by the Ethics Committee of the First Affiliated Hospital of Chongqing Medical University (2020-418). The patients/participants provided their written informed consent to participate in this study.

## Author Contributions

ZL proposed the research direction. JH, SD, and SX were responsible for questionnaire development and data collection. JH was responsible for statistical analysis. JH and SD were responsible for writing the manuscript. ZL was responsible for checking the manuscript. All authors contributed to the article and approved the submitted version.

## Conflict of Interest

The authors declare that the research was conducted in the absence of any commercial or financial relationships that could be construed as a potential conflict of interest.

## Publisher’s Note

All claims expressed in this article are solely those of the authors and do not necessarily represent those of their affiliated organizations, or those of the publisher, the editors and the reviewers. Any product that may be evaluated in this article, or claim that may be made by its manufacturer, is not guaranteed or endorsed by the publisher.
